# Emergency care for older people living with frailty: patient and carer perspectives

**DOI:** 10.1136/emermed-2022-212420

**Published:** 2022-09-06

**Authors:** Emma Regen, Kay Phelps, James David van Oppen, Peter Riley, Jagruti Lalseta, Graham Martin, Suzanne M Mason, Simon Conroy

**Affiliations:** 1 Department of Health Sciences, University of Leicester, Leicester, UK; 2 Emergency Department, University Hospitals of Leicester NHS Trust, Leicester, UK; 3 THIS Institute, University of Cambridge, Cambridge, Cambridgeshire, UK; 4 University of Sheffield, School of Health and Related Research, Sheffield, UK; 5 MRC Unit for Lifelong Health and Ageing, University College London, London, UK

**Keywords:** frailty, qualitative research, emergency care systems

## Abstract

**Background:**

Little is known about how frailty impacts on older people’s experiences of emergency care, despite patient experience being essential to providing person-centred care. This qualitative study reports on the experiences of older people with frailty in the ED and their and their carers’ preferences for emergency care.

**Methods:**

Older people (aged 75+ years) who were at least mildly frail and/or their carers, with current or recent experience of emergency care, were recruited from three EDs in England between January and June 2019. Data were collected via semi-structured in-depth interviews which explored participants’ views on their recent experience of emergency care and their priorities and preferred outcomes. Interviews were audio-recorded, transcribed verbatim and analysed following the principles of the Framework approach.

**Results:**

Forty participants were interviewed: 24 patients and 16 carers who, between them, described ED attendances for 28 patients across the three sites. Often informed by previous negative experiences, there was a strong desire to avoid conveyance to EDs, and a sense of helplessness or acquiescence to attend. Although staff attitudes were on the whole seen as positive, the ED experience was dominated by negative experiences relating to very basic issues such as a lack of help with eating, drinking, toileting and discomfort from long waits on hard trolleys. Participants reported that communication and involvement in decision making could be improved, including involving next of kin, who were viewed as critical to supporting vulnerable older people during sometimes very protracted waits.

**Conclusion:**

Frailty reflects a vulnerability and a need for support in basic activities of daily living, which EDs in this study, and perhaps more widely, are not set up to provide. Changes at the levels of clinical practice and service design are required to deliver even the most basic care for older people with frailty in the ED environment.

**What is already known on this topic**

- A significant proportion of older people are living with frailty.
- Little is known about the impact of frailty on experiences of and preferences for emergency care among older people and their carers.
- This group may have specific needs and expectations which are not currently well understood or being met in busy EDs.

**What this study adds**

- Older people living with frailty wish to be treated with dignity and respect in ED and to receive timely information, communicated clearly and involvement in decision making.
- Preferences for emergency care include a calm and comfortable environment, supported by family with attention to basic physical needs and shorter waiting times.
- EDs in this study, and perhaps more widely, are not set up to provide even the most basic care for older people with frailty in the ED environment.

**How this study might affect research, practice or policy**

- These findings might influence the design of EDs, both in terms of the physical design as well as the facilities and processes provided.

## Introduction

Frailty is a state of vulnerability to poor resolution of homoeostasis after a stressor event and is a consequence of cumulative decline in many physiological systems during a lifetime.^1^ Relatively little is known about the experiences and preferences of older people for emergency care, even less for older people living with frailty. Reviews of existing evidence highlight waiting times, physical and psychological support alongside good communication and information provision in shaping the experiences of older patients in the ED.^2–4^ Although existing studies included people with stereotypical markers of frailty, none used a validated frailty assessment. As such, there is currently limited evidence about whether or how the presence of frailty impacts on older people’s experiences and preferences for emergency care.^4^ These needs must be understood and addressed in order to provide person-centred care.^5^ This qualitative study aimed to provide understanding of the experiences of older people living with frailty in the ED and their and their carers’ preferences for emergency care.

## Methods

### Sample and recruitment

Older people (aged 75+ years) and/or their carers with experience of emergency care were recruited from three EDs in England between January and June 2019. Recruitment was undertaken by experienced research nurses (sites 1 and 2) and clinical staff (site 3) using a purposive recruitment strategy which tried to reflect the population of interest according to: frailty, age, sex, ethnicity, cognitive impairment, place of residence, mode of arrival (ambulance or independent), whether seen in ‘majors’ or ‘minors’, different days of the week and different times of day as summarised below. Participants were included if they were at least mildly frail (≥5 on the Clinical Frailty Scale (CFS)).^6^ Informed consent was taken by staff who recruited participants within 72 hours of patients’ entry to ED, with the majority being recruited on the day of their ED attendance. Recruitment continued until a sample broadly representative of the characteristics above had been interviewed.

### Data collection

Data were collected via semi-structured in-depth interviews using a topic guide informed by the relevant literature^7^ and designed in collaboration with lay co-researchers (non-clinical members of our patient and public forum, who brought a perspective founded in lived experience to the study design and conduct). Interviews explored patient views on their recent experience of emergency care and their priorities and preferred outcomes. Where desired and possible, carers/relatives were also interviewed, alongside or separate from patients according to individual preferences.

In the majority of cases, interviews (conducted by ER or KP) took place within 30 days of the patient’s ED attendance. Most took place in the patient/carer’s usual place of residence—typically their own home, but sometimes the family home, sheltered accommodation or care home. Four interviews, all with carers, took place via telephone at their request. Interviews were audio-recorded and lasted approximately 60 min each.

### Data analysis

All interviews were transcribed verbatim by a professional transcription service and analysed in NVivo using the Framework approach.^8^ Framework is an approach to qualitative analysis designed for applied, often policy-related research, intended to enable systematic, transparent analysis of empirical datasets, often by teams, towards identified practical objectives.^9^ After reading the first transcripts, initially identified themes were used to generate a coding framework; transcripts were coded by two qualitative researchers (ER or KP). Themes generated during the analysis process were discussed and validated by wider members of the research team (SC and/or JDvO) and our lay co-researchers on a regular basis. Data collection and analysis were concurrent, with early findings directing further enquiries in interviews.

### Patient and public involvement

The study was supported by the involvement of two lay co-researchers (PR and JL) who provided advice on recruitment and helped in the design of topic guides and in the analysis. In addition, study updates were shared with the wider Leicester, Leicestershire and Rutland Ageing Patient and Public Involvement Research Forum on a quarterly basis, where useful feedback was provided.

## Results

### Participants and circumstances leading to ED attendance

In total, 40 participants were interviewed: 24 patients and 16 carers who, between them, described ED attendances for 28 patients across the three sites.

Over two-thirds of patients (68%) were female; 43% were aged 75–84 years and 57% were aged over 85 years, including three aged over 95 years. The majority were white British. Twelve had CFS scores of 5, 12 had CFS 6 and 4 had CFS 7. Table 1 shows demographic and ED attendance details for the patients whose ED experiences are described in the study.

**Table 1:** Patient demographics and ED attendance details

	Site 1	Site 2	Site 3	Total
Male	1	3	5	9
Female	7	9	3	19
Aged 75–84 years	2	6	4	12
Aged 85+ years	6	6	4	16
CFS score 5–6	7	9	8	24
CFS score 7	1	3	0	4
White	8	11	8	27
Asian	0	1	0	1
Black	0	0	0	0
ED majors	8	11	7	26
ED minors	0	1	1	2
Lives alone or with spouse	6	9	7	18
Lives with other family members, for example, son/daughter	2	1	1	4
Lives in sheltered accommodation	2	1	0	3
Lives in a care home	0	2	0	2
Lives in own home with live in carers	0	1	0	1
Travelled to hospital by ambulance	8	11	7	26
Travelled to hospital independently/with family	0	1	1	2
Has cognitive impairment	1	1	3	5
Does not have cognitive impairment	7	11	5	23
Attended ED on a weekday	4	12	4	20
Attended ED on a weekend	4	0	4	8
Attended ED in hours (09:00–17:00 Monday–Friday)	2	10	3	15
Attended ED out of hours	6	2	5	13

CFS, Clinical Frailty Scale.

Over a third of patients had a fall as their primary presenting conditions (‘chief complaint’); other common conditions included breathing difficulties, heart problems, stomach/back pain or confusion.

The key themes generated by the analysis of the interviews were an initial reluctance to attend ED, staff care and attitudes, information and communication, environment and comfort and waiting times. These are described in more detail in the sections below.

### Reluctance to attend ED

Most participants were reluctant to be taken to ED. Reasons for this included: previous poor experiences in hospital; only recently having come out of hospital and therefore not wanting to go back in; fear of ‘never coming home again’; fear of a specific hospital; fear of ‘picking up a bug’ and the belief that it would be a ‘waste of time’:

M: I resisted it. Told them [the ambulance crew] that I’d gone through the same thing before. Anyway I went in and my treatment there exactly duplicated the previous time.

I: …So you said you resisted that.

M: Not physically.

I: But you just made it—

M: Yeah, I made it clear that I didn’t want to go…

I: …And why is it that you didn’t want to go in…?

M: Because the last time I went in, they wasted so much time.

In the face of pain, distress and anxiety initial feelings of reluctance and resistance about being taken to ED gave way to feelings of resignation in most cases. In this context, the ‘decision’ to take participants to ED cannot really be seen as a decision at all—certainly not one in which participants could engage in any meaningful way. Ultimately, most accepted the course of action recommended by paramedics who were seen as ‘knowing best’. Family members had, in several cases, played a role in persuading patients that they needed to be taken to ED:

F: So we had to convince my Mom that taking to the hospital.

I: Why did she not want to go?

F: At first she didn’t wanted to but then when she, when we mentioned that was as soon as the treatment is finished we can bring you back home.

I: Right.

F: Everything will be fine and she trusts us of course, so I went into hospital with her in the ambulance. (S2 08C)

### Staff: care and attitudes

Participants generally emphasised attitudes, manner and responsiveness, rather than technical competence, when reflecting on the staff they had encountered in ED. Overall, participants were very positive about ED staff who were seen as being very caring, reassuring and pleasant in their manner:

F1: I didn’t doubt for one minute they cared.

I: Why did you not doubt it?

F1: I don’t know. They kept saying ‘You’ll be all right [name] in a little while’ and they were gently caring. They weren’t sort of saying ‘Well, right …’ and talking amongst themselves as much to say ‘Well right’ you know ‘here comes another one through the machine sort of thing’. But they weren’t. They were genuinely caring. (S1 07PC)

The importance of being treated with respect, particularly in the context of older age, was highlighted. Comments made by some indicated that they had not expected a positive experience—fearing that they would be treated negatively because of their age—but this had not been the case:

F1: I refer to older people, to my age group, normally I’d be the first to say ‘Her age. They haven’t got the patience’. But having been in the last time, well four times, I can’t fault it. (S1 07PC)

A small number of participants, however, did feel that they had been treated negatively due to their age, describing what they saw as a lack of input or intervention during their time in ED/hospital:

I: Right. Okay. It doesn’t sound like much happened whilst you were there?

F: No. It didn’t. To be quite frank with you. They don’t want to know us old ones.

I: Is that how you feel?

F: That’s how I feel…

F: Did you feel the same in every single part of the hospital that you went in?

F: Yeah. They didn’t. If you were over 80 they didn’t want to know. (S2 06P)

A key concern related to staff being unresponsive when patients called for their attention. This was a particular issue for patients who needed assistance with toileting. While highlighted by a relatively small number of patients, this issue had a significantly negative impact on their ED experience—relating as it does to personal comfort and dignity. One patient described how on requesting assistance to go to the toilet she was effectively told it was acceptable for her to soil herself as she was wearing incontinence protection:

M: You get the impression, because you were wearing counter measures

F: Oh—

M: Yeah, proper pants—

I: Incontinence—

M: It’s basically—it’s alright, we’ll sort it out later. Which isn’t very good for dignity.

I: No, it’s not—

F: And I was told 2 or 3 times, when I say I don’t like getting wet underneath—I was told 2 or 3 times oh it doesn’t matter, that’s what the nurses are for.

I: Really?

F: I was more or less told it’s quite alright if you do it in your pants. (S1 09PC)

### Information and communication

The provision of information and effective communication between staff and patients were highlighted as crucial by patients and relatives during their time in ED. Being kept informed of what was happening (tests and treatment), what was likely to happen (ie, admission, transfer or discharge) and some indication of what was wrong was absolutely key. Experiences, however, varied significantly. Some patients reflected positively—staff had kept them informed, explaining pending tests and procedures using a clear and friendly communication style. On the other hand, some participants described negative experiences concerning the provision of information and communication which had caused frustration and bewilderment—as they were left with little sense of what was going on:

I: Did you feel that they were communicating with you what tests had been done and did you get any results back or—?

M: Erm…no, I think it’s just that waiting thing that kills it. So my wife and daughter went off and then at eleven o'clock, two ambulance guys turn up ‘can you get your kit, we’re off to [name of another hospital]’.

I: At what point did you know you were going to be admitted, taken to the [name of other hospital], was that decided quite quickly or did that take quite a while?

M: I think the system knew but I didn’t really…the information really doesn’t go to the patient until it’s happening I think, you know, […] the system seems to know what’s happening, but sometimes the patient doesn’t. (S2 01P)

Linked to the provision of information, some patients commented on the extent to which they had been involved in discussions and decision making about their care. Again, experiences were rather mixed. Some patients reported that they felt listened to and had participated in decision-making; others, however, had not felt involved in important decisions about their care:

I: When did you know that you were going to be moved on to a ward?

F: Not till the stretcher come. I said, ‘Where am I going?’ They said, ‘You’re going on a ward’. I said, ‘What for?’ ‘Oh, we don’t know whether you broke your leg or not’. Well if they didn’t know if I broke my leg or not who did know?

I: So you had had an x ray?

F: Yeah. But nobody ever says, ‘Oh your x ray is fine’ or this that and the other. No. Very badly done.

I: So nobody asked you whether or not you wanted to go onto a ward?

F: No.

I: It was just kind of done without asking you?

F: Without asking me. As though I’m an idiot. I know I’m 82 years but I’ve still got it all up here. (S2 06P)

Together with the provision of information, the manner in which ED staff communicated and the language they used were important to patients and relatives while in ED. Some patients had been impressed by the way in which staff had introduced themselves and explained what they did. Others, however, were not always clear on these details and this was seen as an impediment to communication—patients were often unclear as to whom they had seen and should speak to with queries. A few patients commented that the various different coloured uniforms on display in ED (with no explanation as to what these represented) added to the confusion:

I: Yeah. So you’re in the A&E bit. How many different doctors did you see, do you know?

F: I think I saw two different ones. And some nurses, you don’t know who’s who, to be honest, even when I was in hospital, I said to my husband ‘do you know, there’s so many different colours, you know—

I: Uniforms you mean?

M: Yeah.

F: —one’s got green, one’s got blue and one’s got light blue, one’s got white, one’s got pink’ and so in the end I never knew who really was the Sister and who wasn’t, you know. (S3 05PC)

A few participants commented on the language and communication style used by ED staff—again, painting a rather mixed picture. Some had appreciated the clear, candid and honest language employed by staff when discussing their cases. Others, in contrast, stated that they could not always hear or understand what they were being told, or that things were not always explained properly. Staff, they found, did not always take time to speak slowly and clearly to ensure that information was received and understood.

### Environment and personal comfort

The ED environment created a significant impression on participants’ experiences of emergency care. Several patients at one site in particular drew attention to what they described as an overcrowded, noisy and sometimes rather ‘chaotic’ ED environment:

F: Yeah. And it was, you know, chaotic I thought.

I: Just because of the number of people there or—?

F: Yes, it was a huge amount of people there, seemed to be, you know, and there was somebody calling out ‘I want some biscuits, can you get me some biscuits I want some biscuits!’ and then she turned round and started to sing! And I thought I’ve landed in a mental home! [Laughs]…You know, it sort of went on like that for quite a while. And there was no music, nothing to listen to, except these chaotic things that were happening around me and I thought for crying out loud, you know, it would be nice if somebody turned on some music or if you could watch a screen and, you know—

I: But there was nothing at all.

F: No. (S1 08P)

In contrast, patients at another site, which had recently been refurbished and used glass, rather than curtained cubicles, generally talked about the ED environment in overwhelmingly positive terms. The resulting modern, calm and quieter atmosphere had in turn made them feel calmer as they experienced less rushed and ‘smoother’ care:

F: Well that’s something else, I don’t know—well you know the old A&E—they’re always, in the old A&E was this rustling and bustling about, now there’s a quiet sort of everything’s done equally as quickly but there’s no noise, it’s so quiet and you don’t get this trolleys rushing about and that and handovers and that, they seem to be so easy now, you know, from your ambulance stretcher onto your sort of bed sort of thing, yeah, no, as I say, the handover as I say is good. Everything I found was, it’s done, I don’t know, much smoother—everybody is hurrying about but they don’t seem to be rushing about, you know.

I: So it feels, is it calmer?

F: Oh calmer, definitely. (S2 12P)

Comments regarding privacy while in ED were, on the whole, quite positive. Several patients who had experienced the new glass cubicles in the refurbished site felt that these had resulted in improved privacy. Interestingly, even those patients who described care in very busy and overcrowded conditions in other EDs did not raise privacy as a concern even though the systems employed by staff to maintain privacy appeared to be far from ideal:

F: No, no. But it was funny because, as I said before, if you needed to be examined privately, if a doctor wanted to, they’d yank somebody out of this cubicle, push them in, shut the curtain, the doctor would do what he’d got to do, the curtain would come out again and then somebody had gone in! And it was funny I suppose but—

I: How did you feel in terms of kind of privacy and dignity and things like that in this ridiculous situation?!

F: Well, er, you have a blanket over you so…and they don’t do anything, they only take your blood pressure in view of everybody else, you know, everybody, they take you into one of these little cubicles— (S1 06P)

Being comfortable and having basic physical needs met were highlighted as having an important impact on participants’ ED experience. There were two main aspects to this: the availability of food and drink; and the comfort of hospital trolleys/beds. Having access to food and drink while waiting in ED (often for considerable periods of time) was an important element of patients’ and relatives’ well-being and personal comfort. Some patients reported very positive experiences in this regard, with ED staff proactively offering some kind of drinks and/or light refreshments which were very much appreciated:

I: Right. And you hadn’t…had anybody got you up and moved you or asked you if you needed the toilet or anything like that?

F: Oh yes they came and did…they gave me a buzzer, so said if you need the toilet or anything, just buzz. They did make me a drink and brought me a sandwich, because I hadn't had anything since breakfast. (S2 04P)

Other participants, however, including several with diabetes, reported that staff had not enquired as to whether they would like something to eat or drink and did not offer anything. Some participants said that they had been given refreshments when they asked for them, but one relative had to make their own arrangements:

F: Not this time, but the time before, I actually went up to one of the nurses and said, ‘Is there any possibility of organising something for my husband to eat, he’s dia-’. ‘Oh we don’t usually’. I said, ‘Well he’s diabetic and he’s not ate for’, whatever it was, six hours or whatever. She said ‘well there’s a café’, is it Costa, whatever it is.

I: Oh right, OK. So they, as a matter of course, no matter how long someone’s in the Emergency Department they won’t give them anything to eat?

F: Well I don’t—

I: That was the message that you received?

F: Yeah. (S3 06PC)

Several participants described very uncomfortable and even painful hours lying on hospital trolleys or beds unable to sleep or rest because of the discomfort. The problem was exacerbated by the fact that patients often had to wait in ED for many hours. Trolleys and beds were described as being too hard, too small and very uncomfortable:

M: See this is the concern I’ve got about it, is that there’s this target of you know, once you get into A&E you’ll be in a bed in four hours. Well, I suppose technically they meet that when they put you into a cubicle. But that’s certainly not a bed, and I wouldn’t recommend it. There’s no way I slept in that. I mean I’m only five feet ten, I’m not huge. There’s no way I can lie in that bed comfortably, no matter what they do. I would imagine that if I’m five feet ten, there’s only about five feet six of that bed.

I: So it’s uncomfortable.

M: It’s uncomfortable. (S1 10PC)

The importance of having family members present during an ED attendance was highlighted by interviewees. Just over half of patients had someone with them in ED—typically spouses or children—while approximately one-third reported that they were alone during this time (information was missing in the remaining cases). Family members provided a source of practical and emotional support during what could otherwise be a quite daunting experience. In contrast, some of those on their own in ED described the experience as frightening, lonely or simply boring:

I: Did you feel safe?

F: Yes, I felt safe, yes. I wasn’t upset or anything.

I: No, you weren’t frightened or worried or anxious?

F: No, I don’t think I was because [name of grand-daughter] was with me, if she hadn’t have been I probably would have been.

I: Right, OK, yeah. So it was important to have somebody with you.

F: That’s the way I felt, yes. (S1 05P)

### Time waiting in ED

The issue of waiting times in ED emerged as a prominent theme during interviews. Just over a quarter of patients had waited 4 hours or less in ED before being admitted, transferred or discharged. These participants expressed satisfaction and sometimes surprise at such ‘swift’ treatment. In contrast, another quarter reported they had waited in ED approximately 12 hours or longer before being admitted to a ward. Such long waits were difficult to endure, especially when combined with having to lie on uncomfortable trolleys/beds and going without food and drink for extended periods of time:

F: Oh yes. The first time I ever went in there, oh boy, I’ve never got over it, my bum is still suffering from that first time.

I: Really.

F: Yeah.

I: Were you on like one of those narrow trolley things?

F Yeah.

I: Oh dear. And were you on there for a long time?

F: Oh I was, fifteen hours before they put me into a bed…

The first time I went and I were there for fifteen hours before they found me a bed but, apart from that, I have no complaints. (S1 04P)

Comments made by some relatives during interviews provided insight into the impact long waits in ED could have on them as well as their loved ones. They were often older people themselves and had also experienced discomfort, difficulties obtaining refreshments and lack of sleep:

F: Well I’ve been very well but I think visiting [name of husband] for five weeks and the first two days I spent 60 hours without going to bed—

I: Oh my goodness.

F: —simply because he went in as an emergency— … and I sat all night with him while they were waiting for the consultant to have a look at him and then we went to the [name of another hospital] for the next night and I sat all night there as well. (S2 09C)

## Discussion

This study described the emergency care experiences and preferences of older people living with frailty (and their carers). People wished to be treated by caring, responsive staff and with respect, which was understood in terms of dignity and appropriate care. The provision of timely information communicated clearly and honestly to both patients and relatives was also highlighted, not least in enabling patients to be involved in decision-making about their own care. Participants wanted to experience emergency care in a calm, quiet environment where basic needs for privacy, comfort and food/drink were met, and where they could be supported by relatives and friends. The desire for shorter waiting times was also prominent. Study participants described very mixed and sometimes rather negative experiences in terms of whether these preferences were actually met. This perhaps explains the reluctance expressed by many about attending hospital in the first place. In this study, older people living with frailty accessing emergency care had often very negative experiences as even their most fundamental needs were not consistently met. Notably absent was any sense of aspiration for anything beyond basic needs to be addressed. In the language of Maslow, belonging, esteem and self-actualisation were remote, abstract concepts in the face of basic needs remaining unmet.

For older people living with frailty, interactions with staff are inextricably linked to their own sense of self and dignity. Being treated with respect and kindness provides validation and legitimacy, whereas being ignored or dismissed (eg, when seeking help with toileting) can have a lasting negative impact on self-esteem.^2^ Similarly with regard to information and communication, older people living with frailty may need particular support to be involved in decision making,^10^ such as timely information communicated clearly, and for staff to check that patients have understood.^11^ Communication barriers relating to hearing/visual problems, language or cognitive impairment need to be identified and overcome.^3^ The presence, involvement and advocacy of relatives is imperative for both older people living with frailty and their carers.^11 12^

Our findings show that the noisy, busy ED environment poses a particular challenge to older people living with frailty who prefer to receive care in a calm and quiet setting. They appear to be particularly sensitive to and distressed by the physical privations they often experience in ED in the form of uncomfortable trolleys and a lack of food and drink. While previous studies remark on the ‘resilience’ or ‘tolerance’ demonstrated by older people encountering long waits in ED,^13 14^ this may be more challenging for older people with frailty. Participants reflected very negatively on this element of their experience; they had no choice but to ‘tolerate’ long and/or uncomfortable waits which took a considerable toll on them at the time and appeared to make them more reluctant to attend the ED when faced with future emergency episodes.

The findings reported from this study are consistent with previous research on the experiences of emergency care for older people which emphasise the importance of information and communication, staff attitudes, waiting times and physical/environmental needs in shaping those experiences.^2–4 15–18^ These aspects of care have also been identified as instrumental in determining the experiences of adult patients generally in the ED.^19–21^ In many respects, the needs and priorities of older people living with frailty in the ED mirror those of patients of all ages, not least in the import attached to relational aspects of care (communication, compassion and empathy) over technical competence.^19–21^ Our findings suggest, however, that these needs and preferences are *particularly pressing* for older people living with frailty as they are more vulnerable in the ED environment both physically and emotionally when these needs are not met.^4^ While our findings echo previous studies, the use of the frailty construct provides a helpful anchor against which specific links to practice changes can be proposed. For example, NHS policy is to assess for frailty in older people presenting to emergency care; the presence of moderate-to-severe frailty identifies a vulnerable group with needs who can benefit from enhanced care (described below).

### Strengths and limitations

Participants included those with mild-to-severe frailty and so we are able to report the views of a group of patients who have previously been excluded from research in this area, yet represent a sizeable and growing number of those accessing emergency care. Although views on desirable outcomes from ED attendance are not explored here, these data were collected during the study and are presented separately.

Despite the efforts of recruiting staff, the lack of ethnic diversity within our sample is a limitation. We did not recruit any patients from the very severe frailty categories (CFS 8 and 9). Furthermore, we were unable to explore whether there were any relationships or associations between different levels of frailty (ie, mild/moderate/severe) and particular experiences and preferences for emergency care. This would require a larger, possibly mixed methods study, including more patients within each frailty stratum. Although most interviews were conducted within 30 days of the ED attendance, there is always the possibility of inaccurate recall. Moreover, the focus on relational aspects of care may reflect the purview of both patients and interviewers who, having relatively little knowledge of medical processes and procedures, focused on interpersonal elements of the ED experience.^19^

### Implications for policy and practice

This study provides important evidence about the extent to which frailty impacts on older people’s experiences of emergency care. Our research suggests that frailty can result in a particular vulnerability in ED if physical (ED environment, personal comfort, waiting) and emotional (sense of dignity, communication, involvement, family support) needs are not met.

**Figure 1:**
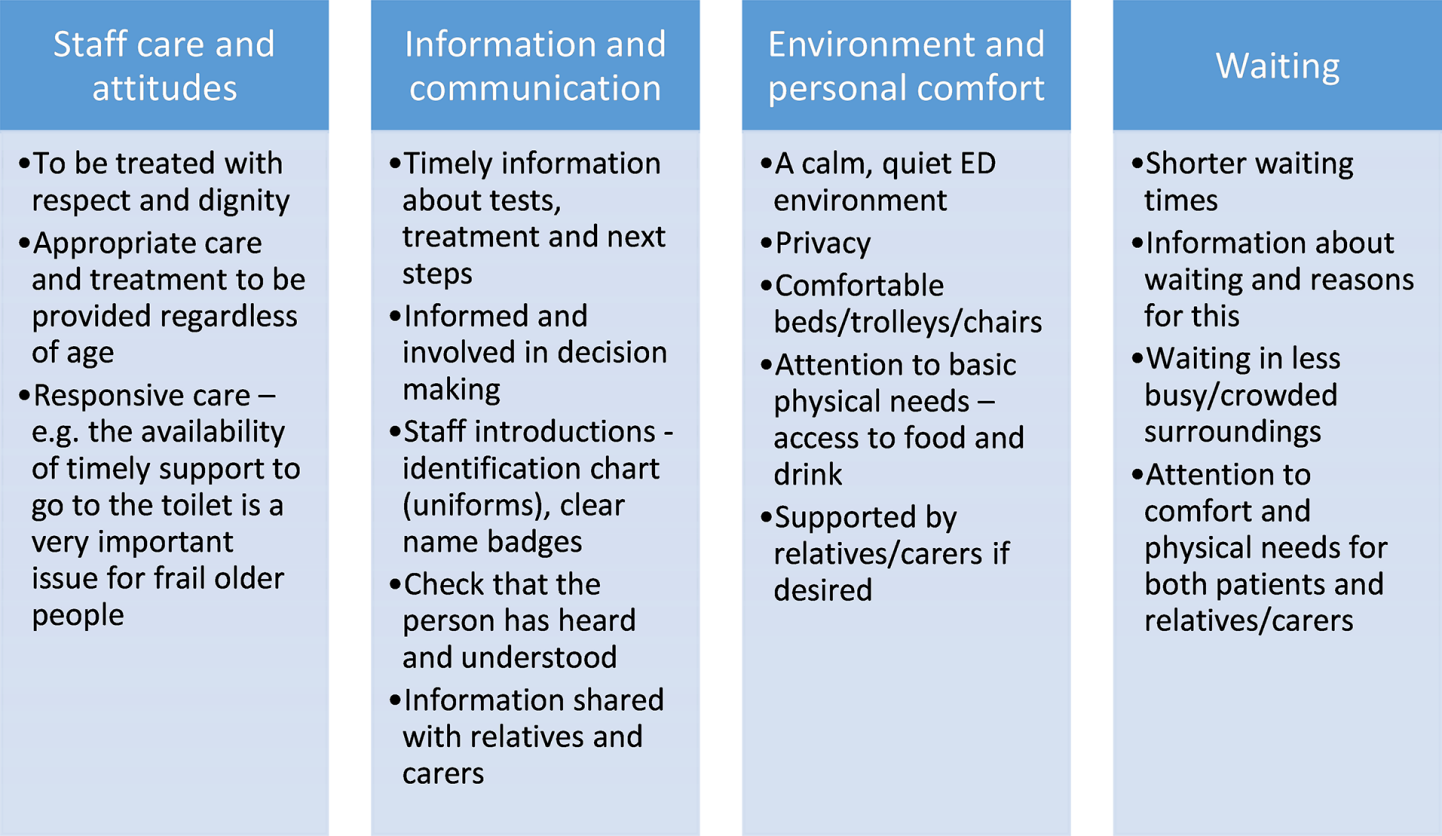
Meeting the needs of trail order people in ED: recommendations for practice.

Policy (eg, Ageing Well within the NHS England Long Term Plan^22^) encourages the delivery of person-centred care, including ‘working with the person’s values and belieefs’ and ‘sharing decision making’.^5^ Yet if basic needs cannot be met, it is difficult to see how higher-level person centredness can be achieved. At the level of practice, we make several recommendations which might potentially be delivered in clinical practice (figure 1). In particular, recommendations relating to staff care and attitudes and information and communication could make a significant positive difference to the experiences of older people living with frailty in ED. While the ED environment and waiting times may be harder to change, healthcare professionals can help older people living with frailty by being mindful of their comfort, physical needs, the flow of information and the importance of patient/carer involvement. Indeed, in an environment where waiting times may be extending,^23^ the importance of a person-centred environment becomes even greater.

More broadly and given the challenges of more fundamental changes to the fabric of the ED and the pressures on this part of the healthcare system, policy makers and practitioners need to consider service development changes when responding to the needs of older people living with frailty requiring urgent and emergency care. These may include the adoption of new approaches closer to the hospital ‘front door’, interventions which minimise or even bypass the time these patients need to spend in ED as well as frailty friendly design in ED.^24 25^ Any new models of care must be based on robust research evidence which should relate to clinical and cost-effectiveness and to patient and carer perspectives.^26^

## Data Availability

All data relevant to the study are included in the article or uploaded as supplementary information. The quotes (data) are included in the text, but the transcripts are not available given the risk of breach of confidentiality.
